# Comparing a Data Entry Tool to Provider Insights Alone for Assessment of COVID-19 Hospitalization Risk: Pilot Matched Cohort Comparison Study

**DOI:** 10.2196/44250

**Published:** 2023-11-16

**Authors:** Gezan Yahya, James B O'Keefe, Miranda A Moore

**Affiliations:** 1 Rollins School of Public Health, Emory University Atlanta, GA United States; 2 Department of Family and Preventive Medicine, School of Medicine, Emory University Atlanta, GA United States; 3 Department of Medicine, School of Medicine, Emory University Atlanta, GA United States

**Keywords:** COVID-19, risk assessment, hospitalization, outpatient, telemedicine, data, tool, risk, assessment, utilization, algorithm, symptoms, disease, community, patient, decision making tool, risk algorithm

## Abstract

**Background:**

In March 2020, the World Health Organization declared COVID-19 a global pandemic, necessitating an understanding of factors influencing severe disease outcomes. High COVID-19 hospitalization rates underscore the need for robust risk prediction tools to determine estimated risk for future hospitalization for outpatients with COVID-19. We introduced the “COVID-19 Risk Tier Assessment Tool” (CRTAT), designed to enhance clinical decision-making for outpatients.

**Objective:**

We investigated whether CRTAT offers more accurate risk tier assignments (RTAs) than medical provider insights alone.

**Methods:**

We assessed COVID-19–positive patients enrolled at Emory Healthcare's Virtual Outpatient Management Clinic (VOMC)—a telemedicine monitoring program, from May 27 through August 24, 2020—who were not hospitalized at the time of enrollment. The primary analysis included patients from this program, who were later hospitalized due to COVID-19. We retroactively formed an age-, gender-, and risk factor–matched group of nonhospitalized patients for comparison. Data extracted from clinical notes were entered into CRTAT. We used descriptive statistics to compare RTAs reported by algorithm–trained health care providers and those produced by CRTAT.

**Results:**

Our patients were primarily younger than 60 years (67% hospitalized and 71% nonhospitalized). Moderate risk factors were prevalent (hospitalized group: 1 among 11, 52% patients; 2 among 2, 10% patients; and ≥3 among 4, 19% patients; nonhospitalized group: 1 among 11, 52% patients, 2 among 5, 24% patients, and ≥3 among 4, 19% patients). High risk factors were prevalent in approximately 45% (n=19) of the sample (hospitalized group: 11, 52% patients; nonhospitalized: 8, 38% patients). Approximately 83% (n=35) of the sample reported nonspecific symptoms, and the symptoms were generally mild (hospitalized: 12, 57% patients; nonhospitalized: 14, 67% patients). Most patient visits were seen within the first 1-6 days of their illness (n=19, 45%) with symptoms reported as stable over this period (hospitalized: 7, 70% patients; nonhospitalized: 3, 33% patients). Of 42 matched patients (hospitalized: n=21; nonhospitalized: n=21), 26 had identical RTAs and 16 had discrepancies between VOMC providers and CRTAT. Elements that led to different RTAs were as follows: (1) the provider *“missed”* comorbidity (n=6), (2) the provider noted comorbidity but undercoded risk (n=10), and (3) the provider miscoded symptom severity and course (n=7).

**Conclusions:**

CRTAT, a point-of-care data entry tool, more accurately categorized patients into risk tiers (particularly those hospitalized), underscored by its ability to identify critical factors in patient history and clinical status. Clinical decision-making regarding patient management, resource allocation, and treatment plans could be enhanced by using similar risk assessment data entry tools for other disease states, such as influenza and community-acquired pneumonia. The COVID-19 pandemic has accelerated the adoption of telemedicine, enabling remote patient tools such as CRTAT. Future research should explore the long-term impact of outpatient clinical risk assessment tools and their contribution to better patient care.

## Introduction

In March 2020, the World Health Organization declared the COVID-19 outbreak a pandemic [1]. Since its emergence from Wuhan, China [2], COVID-19 has spread rapidly, having overwhelmed health care systems and causing over 2 million deaths [3]. With the ongoing COVID-19 pandemic, there is an urgency to understand who is most at risk for severe COVID-19 in order to guide health care resource allocation, usage, and management of care [4]. As hospitalization rates of people with COVID-19 increase with age and underlying medical conditions [5-8], early monitoring and care are needed for patients at high risk. Furthermore, as outpatient treatments become available, targeting high-risk patients remains a challenge [9].

Usage of a risk prediction algorithm for future hospitalization of outpatients with COVID-19 is not well explored but is critical to facilitate clinical decision-making. A risk prediction tool can assist health care providers in their clinical decision-making and can impact patient self-management and treatment decisions [10]. Although providers understand the logic of a risk prediction algorithm, accurately applying the algorithm in a fast-paced clinical visit may be challenging. Usage of a point-of-care data entry tool based on the algorithm may lead to a risk assignment with a higher fidelity to the algorithm.

To help health care providers in their decision-making, a point-of-care data entry tool, “COVID-19 Risk Tier Assessment Tool” (CRTAT), was created to assign an estimated level of risk (“risk tier”) for future hospitalization for outpatients with acute COVID-19. We aim to determine whether the CRTAT has a higher fidelity to risk for future hospitalization assessment algorithms (ie, it assigns a more appropriate risk tier) than medical provider insights alone. This study will establish a new method to facilitate and enhance clinical decision-making of patients with COVID-19 and will provide greater insight into the effectiveness of using a point-of-care data entry tool to apply a disease risk algorithm.

## Methods

### Recruitment

In March 2020, Emory Healthcare created a Virtual Outpatient Management Clinic (VOMC) for nonhospitalized adult patients with COVID-19 in Atlanta, Georgia, offered to all Emory Healthcare patients who screened positive for COVID-19. The VOMC was a telemedicine clinic staffed by primary care physicians and advance practice providers (APPs). The VOMC intake team included 14 physicians and 3 APPs from primary care clinics, and the VOMC follow-up call teams included 19 redeployed registered nurses and 20 APPs. VOMC providers were available to any patient at home with acute COVID-19. VOMC providers completed an initial intake assessment via telehealth consultations, assigned patients an estimated “tier” of risk for future hospitalization using an Emory Healthcare–created prediction algorithm (Table 1) [11], and provided regular follow-up calls until patients experienced improvement in symptoms [11,12]. All intake providers were trained in the use of the risk assessment tool in a 1-hour webinar.

**Table 1:** Emory Healthcare COVID-19 Virtual Outpatient Management Clinic's risk assessment algorithm.

	Tier 1 (low risk)	Tier 2 (intermediate risk)	Tier 3 (high risk)
Patient characteristics	Age <60 years (no risk factors)	Age <60 years with 1-2 moderate risk factors^a^; age 60-69 years with no risk factors or 1 controlled moderate risk factor; pregnant patients	Age ≥70 years; age <70 years with 1 or more high risk factors^b^; age 60-69 years with 1 or more moderate risk factors; age <60 years with 3 or more moderate risk factors
Symptoms	Nonspecific, upper respiratory tract infection, or cough	Nonspecific, upper respiratory tract infection, or cough	Severe cough, DOE^c^, wheezing, and chest tightness
Course	Stable (if within the first 6 days) or improving	Stable (if within the first 6 days) or improving; otherwise, tier 1 patient without improvement after 6 days	Any new or worsening lower respiratory tract infection symptoms; otherwise, tier 2 patient without improvement after 6 days; or nonrespiratory complication or decompensated chronic condition
Support system	Able to self-isolate; adequate support	Otherwise tier 1, but uncertain support	Otherwise lower tier, but unstable support system

^a^Moderate risk factors include a BMI between 30 and 39 kg/m^2^, asthma, chronic kidney disease, diabetes mellitus, hypertension, pregnancy, and smoking (past or current).

^b^High risk factors include a BMI of >40 kg/m^2^, metabolic syndrome, cirrhosis, cardiovascular disease, chronic obstructive pulmonary disease, end-stage renal disease, immunocompromised status, frailty, and living in a health care facility.

^c^DOE: dyspnea on exertion.

Emory Healthcare’s COVID-19 hospitalization risk prediction algorithm, based on known risk factors from prior studies (including patient-specific characteristics, comorbidity, current illness severity, recent clinical course, and social factors), was a pragmatic approach allowing follow-up call teams to focus efforts on individuals at the highest risk of severe illness and hospitalization. During this study period, providers collected patient information during the intake VOMC visits, entering the information in the clinical note in real time. Patients were assigned to risk tiers 1-3 by the provider upon completion of the VOMC intake visit using a single-page decision tool. Tier 1 was defined as “low risk,” tier 2 as “intermediate risk,” and tier 3 as “high risk.” Patients assigned to tier 1 had to meet all of the following criteria: age of <60 years; no comorbidities known to increase the risk of severe COVID-19; no lower respiratory tract symptoms, except for mild cough; and ability to self-isolate [11]. Patients assigned to tier 2 included adults aged 60-69 years without comorbidities and adults aged <60 years with moderate-risk comorbidities or with symptoms persisting in the second week of their illness [11]. Patients assigned to tier 3 included adults meeting any of the following criteria: age of 70 years, younger age with a specific high-risk comorbidity or multiple comorbidities, new or worsening lower respiratory tract symptoms, or uncertain ability to self-isolate [11]. Subsequent studies revealed that these tiers were associated with hospitalization rates of 1%, 7%, and 23%, respectively [11]. Further details of Emory Healthcare’s COVID-19 hospitalization risk prediction algorithm and tiering system have been previously described [11].

The CRTAT was designed using Qualtrics (Qualtrics), and is a web-based survey tool that enables providers to input patient information to determine risk tier based on the Emory Healthcare risk algorithm, using mainly if/then logic (Multimedia Appendix 1).

A retrospective review of medical records was performed for outpatients with nasopharyngeal sampling–confirmed COVID-19 screened at the VOMC. Eligibility criteria included the following: (1) COVID-19 diagnosis by polymerase chain reaction using nasopharyngeal swab samples, (2) being in isolation at the time of diagnosis, (3) being enrolled at a VOMC during the period of May 27 through August 24, 2020, and (4) having been hospitalized for COVID-19. Exclusion criteria were hospitalization when the results of the polymerase chain reaction test were obtained or prior to VOMC enrollment.

### Statistical Analysis

The intervention group included all adult patients who were hospitalized for COVID-19. The control group included patients who were not hospitalized for COVID-19 and were matched with those who were hospitalized for COVID-19. Matching was performed through pairwise combination using Stata/SE 16 software (Statworks Group) and was based on age, gender, and risk factors including obesity, asthma, chronic kidney disease, diabetes, hypertension, pregnancy, lung disease, cancer, cardiovascular disease, and immunosuppression.

Data were obtained from the initial patient intake assessment notes in Emory’s clinical data warehouse. The data extracted included patient demographics, comorbidities, presenting symptoms, social factors, and initial risk tier assignment (RTA). The data were entered into the CRTAT to produce an algorithm-defined future hospitalization risk tier. The risk tiers produced by the CRTAT were compared with those assigned during the patient intake visit by VOMC providers. Data entry and extraction were performed by a member of the research team and were completed in 1 day. Little training was provided to the data analyst given their nonclinical background and role in the study. Descriptive statistics were determined using Stata/SE 16 software [13]. Potential elements that led to differences were identified through a retrospective review of medical records by an Emory Healthcare provider trained in the COVID-19 risk prediction algorithm.

### Ethical Considerations

This study was deemed to not be human subjects research by the Emory Institutional Review Board; hence, no informed consent was needed.

## Results

The study included 21 patients who were hospitalized for COVID-19 (intervention group) and 21 patients who were not (control group; Table 2). As we matched the nonhospitalized sample to the hospitalized sample by age, gender, and risk factors, the samples displayed the same distributions. Our population was mostly younger than 60 years (14, 67% hospitalized and 15, 71% nonhospitalized) with ≥1 moderate risk factors (hospitalized group: 1 among 11, 52% patients; 2 among 2, 10% patients; and ≥3 among 4, 19% patients; nonhospitalized group: 1 among 11, 52% patients, 2 among 5, 24% patients, and ≥3 among 4, 19% patients). Overall, high risk factors were prevalent in 45% (n=19) of the sample (ie, cancer, cardiovascular disease, chronic kidney disease, and immunosuppression), 52% (n=11) of the hospitalized sample, and 38% (n=8) of the nonhospitalized sample. Approximately 83% (n=35) of the sample reported nonspecific symptoms; 60% (n=25) of them reported symptoms of upper respiratory tract infection, 36% (n=15) of them reported a minor cough, 55% (n=23) of them reported other symptoms of lower respiratory tract infection, and 88% (n=37) reported no mental health symptoms. Symptoms were generally mild (hospitalized: 12, 57% of patients; nonhospitalized: 14, 67% of patients). Most patients (n=19, 45%) were seen on days 1-6 of their illness with a stable symptom course over these 1-6 days (hospitalized: 7, 70% of patients; nonhospitalized: 3, 33% of patients).

**Table 2:** Characteristics of patients with COVID-19 (N=42) in the Emory Healthcare Virtual Outpatient Management Clinic between May 27 and August 24, 2020.

Factor				Overall sample, n (%)		Hospitalized patients, n (%)		Nonhospitalized patients, n (%)
**Age (years)**								
	<60			29 (69)		14 (67)		15 (71)
	60-69			9 (21)		5 (24)		4 (19)
	≥70			4 (10)		2 (10)		2 (10)
**Moderate risk factors**								
	None			5 (12)		4 (19)		1 (5)
	1			23 (55)		11 (52)		11 (52)
	2			7 (17)		2 (10)		5 (24)
	≥3			8 (19)		4 (19)		4 (19)
**High risk factors (ie, cancer, cardiovascular disease, chronic kidney disease, and immunosuppression)**								
	No			23 (55)		10 (48)		13 (62)
	Yes			19 (45)		11 (52)		8 (38)
**Nonspecific symptoms**								
	No			7 (17)		3 (14)		4 (19)
	Yes			35 (83)		18 (86)		17 (81)
**Upper respiratory tract infection symptoms**								
	No			17 (41)		8 (38)		9 (43)
	Yes			25 (60)		13 (62)		12 (57)
**Lower respiratory tract infection symptoms**								
	None			4 (10)		2 (10)		2 (10)
	Minor cough			15 (36)		7 (33)		8 (38)
	Other (ie, chest tightness or dyspnea on exertion)			23 (55)		12 (57)		11 (52)
**Mental health symptoms**								
	No			37 (88)		18 (86)		19 (91)
	Yes			5 (12)		3 (14)		2 (10)
**Symptom severity**								
	Mild			26 (62)		12 (57)		14 (67)
	Moderate			13 (31)		9 (43)		4 (19)
	Severe			2 (5)		0 (0)		2 (10)
	N/A^a^			1 (2)		0 (0)		1 (5)
**Symptom day**								
	1-6			19 (45)		10 (48)		9 (43)
	15-21			10 (24)		5 (24)		5 (24)
	7-14			12 (29)		6 (29)		6 (29)
	N/A			1 (2)		0 (0)		1 (5)
**Symptom course days 1-6**								
	**Days 1-6**							
		Improving	6 (32)		3 (30)		3 (33)	
		Stable	10 (53)		7 (70)		3 (33)	
		Worsening	3 (16)		0 (0)		3 (33)	
	**Days 7-14**							
		Improving	2 (17)		0 (0)		2 (33)	
		Stable	7 (58)		3 (50)		4 (67)	
		Worsening	3 (25)		3 (50)		0 (0)	
	**Days 15-21**							
		Improving	5 (50)		1 (20)		4 (80)	
		Stable	5 (50)		4 (80)		1 (20)	
		Worsening	0 (0)		0 (0)		0 (0)	
	**Day 22 and beyond**							
		N/A	42 (100)		21 (100)		21 (100)	

^a^N/A: not applicable.

A comparison of the RTAs between the VOMC providers and the CRTAT revealed 26 identical RTAs (tier 1: n=1, tier 2: n=4, and tier 3: n=21) and 16 different RTAs. Most hospitalized patients and all nonhospitalized patients with noncongruent RTAs (n=15) were assigned a lower tier by the provider. One patient assigned a higher RTA by the provider was assigned a lower RTA in the CRTAT due to mild and stable symptoms on day 16 from onset (Table 3).

**Table 3:** Comparison of risk tier assignments between Virtual Outpatient Management Clinic providers and the COVID-19 Risk Tier Assessment Tool for patients (N=42) enrolled in the study at the Emory Healthcare Virtual Outpatient Management Clinic between May 27 and August 24, 2020.

Provider risk tier assignment		COVID-19 Risk Assessment Tool’s risk tier assignment, n		
		1	2	3
**All patients**				
	1	1	3	1
	2	0	4	11
	3	0	1	21
**Hospitalized patients (n=21)**				
	1	0	0	1
	2	0	3	5
	3	0	1	11
**Nonhospitalized matched (control) patients (n=21)**				
	1	1	3	0
	2	0	1	6
	3	0	0	10

A review of medical records revealed elements that led to different RTAs. First, VOMC providers *“missed”* comorbidities (6 patients); providers either did not record comorbidities during the intake visit or did not code the comorbidity as a risk. Second, VOMC providers recorded comorbidities but assigned a lower risk tier (10 patients); for example, VOMC providers documented a patient as having asthma and immunosuppression from HIV but assigned the patient to tier 2 instead of tier 3. Third, VOMC providers noted a patient’s symptom severity and course but assigned a lower risk tier (7 patients); for example, VOMC providers documented a patient as having moderate and worsening symptoms and assigned the patient to tier 2 instead of tier 3.

## Discussion

### Principal Findings

Our findings suggest that the CRTAT has higher fidelity in its risk tier assessment algorithm than do medical provider insights alone. These findings support the use of a point-of-care data entry tool in clinical practice to assign a level of risk for future hospitalization for patients with COVID-19.

In this pilot study, our CRTAT assigned many patients to higher risk tiers than VOMC providers. While the CRTAT more appropriately categorized hospitalized patients, we acknowledge that the use of the tool will increase assignment of patients to a higher risk tier based on our matched sample’s outcomes.

### Limitations

The study sample was small due to the time burden of manual extraction of medical records. Additionally, the study was limited to a single institution. The patients enrolled in the study were seen at the VOMC and are not representative of all patients diagnosed with COVID-19 in Atlanta, Georgia. All intake providers who assessed VOMC patients and assigned risk tiers were trained in the use of the risk assessment algorithm and do not represent all providers who provide care to patients with COVID-19. Therefore, our results may not be generalizable to other provider populations. Further, our study does not explore the limitations of integrating these decision-aid tools into the clinical workflow or the electronic health record.

### Comparison With Prior Work

Risk calculators and risk stratification tools are widely used in clinical practice to estimate risk for severe diseases or determine the need for hospitalization among individuals. Common risk scores include the Wells Criteria [14]—a clinical decision-making rule used to estimate the probability of acute pulmonary embolism in patients—and the Patient Outcomes Research Team score [15,16]—a rule used to estimate morbidity, mortality, and need for hospitalization in adults with community-acquired pneumonia.

While there are existing risk scoring systems, there are no current risk prediction data entry tools developed for use in telemedicine. For COVID-19, proposed risk scores are shown to be at high risk of bias, and several risk scores depend on values that cannot be collected in a telemedicine visit, including vital signs and laboratory values [10]. Our CRTAT is unique because it is a telemedicine risk assessment tool designed to assign a level of risk for future hospitalization without collecting laboratory values or vital signs. The CRTAT is well suited for outpatient collection of self-reported information, risk assessment, and clinical decision-making.

### Future Directions

Use of decision support tools in medicine is shown to improve clinical decision-making and support delivery of quality care [17]. As telemedicine is becoming an established care delivery model for acute diseases due to the COVID-19 pandemic, use of a data entry tool to predict the risk of severe illness has wide-reaching applications in clinical practice. Applying a telemedicine risk prediction tool to other diseases such as influenza and community-acquired pneumonia could result in better clinical outcomes for patients.

Previous studies have shown that telemedicine and remote patient monitoring are associated with better clinical outcomes including fewer emergency room visits and hospitalizations [18]. More research is needed to highlight the long-term impacts of using telemedicine risk assessment tools.

### Conclusions

Our CRTAT has higher fidelity in its algorithm to assess the risk for future hospitalization than do medical provider insights alone, primarily by identifying high-risk features in patient history and clinical status. CRTAT demonstrates how technology can be harnessed to enhance patient care. Accurately predicting the risk of severe disease or hospitalization in general outpatient visits could lead to more appropriate monitoring of outpatients and clinical interventions, leading to better long-term health outcomes. Additionally, such tools could lead to better allocation of health care resources in general.

## Appendix

Multimedia Appendix 1 COVID-19 Risk Tier Assessment Tool.

## Abbreviations

APP: advance practice provider
CRTAT: COVID-19 Risk Tier Assessment Tool
RTA: risk tier assignment
VOMC: Virtual Outpatient Management Clinic

## Data Availability

The data sets generated or analyzed during this study are available from the corresponding author on reasonable request.
