# Supplementary figures and images for: Comparing a Data Entry Tool to Provider Insights Alone for Assessment of COVID-19 Hospitalization Risk: Pilot Matched Cohort Comparison Study
Source: JMIR Form Res. 2023 Nov 16;7:e44250. doi: 10.2196/44250 (PMC10691529; doi:10.2196/44250)

**Appendix 1.** COVID-19 Risk Tier Assessment Tool


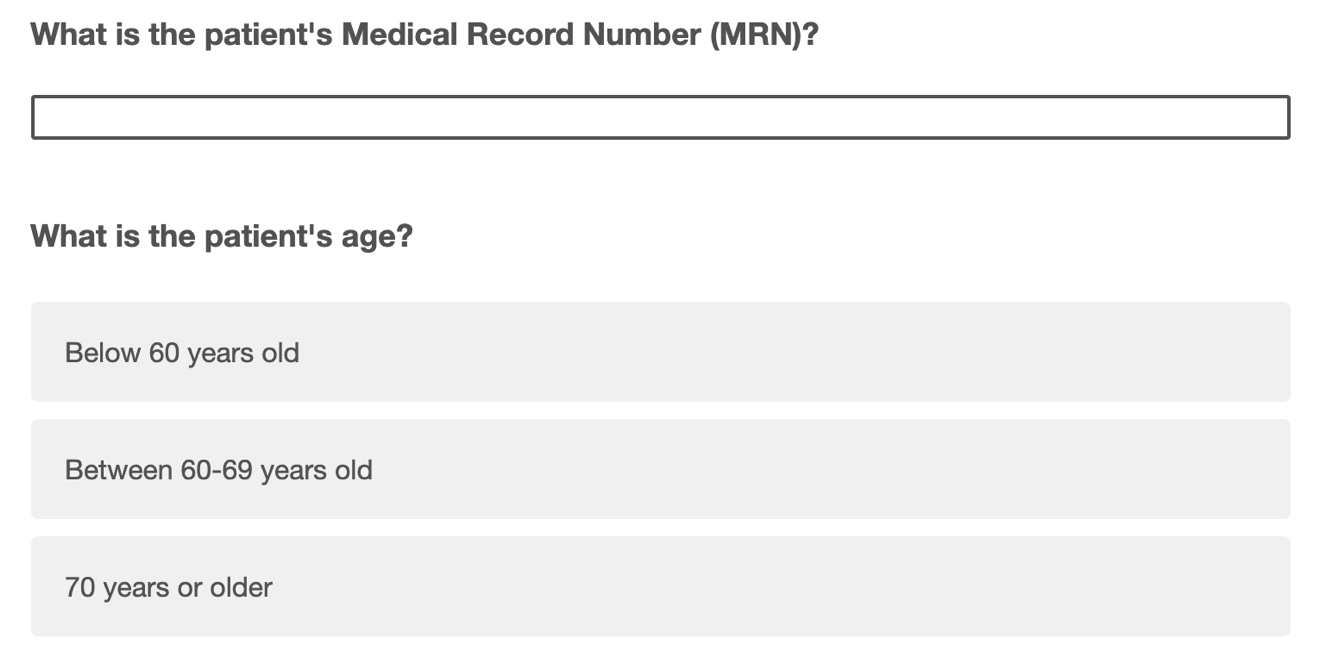


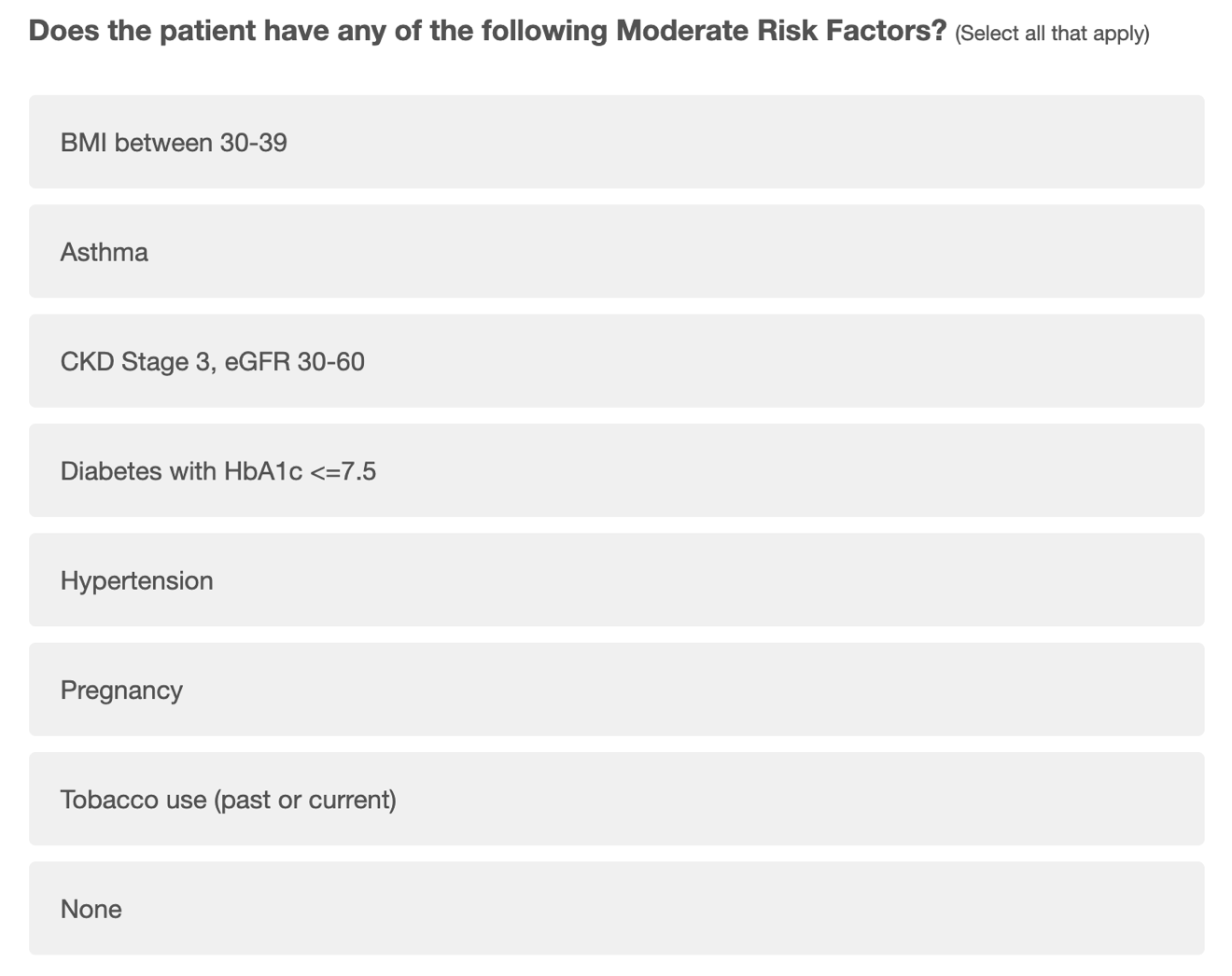


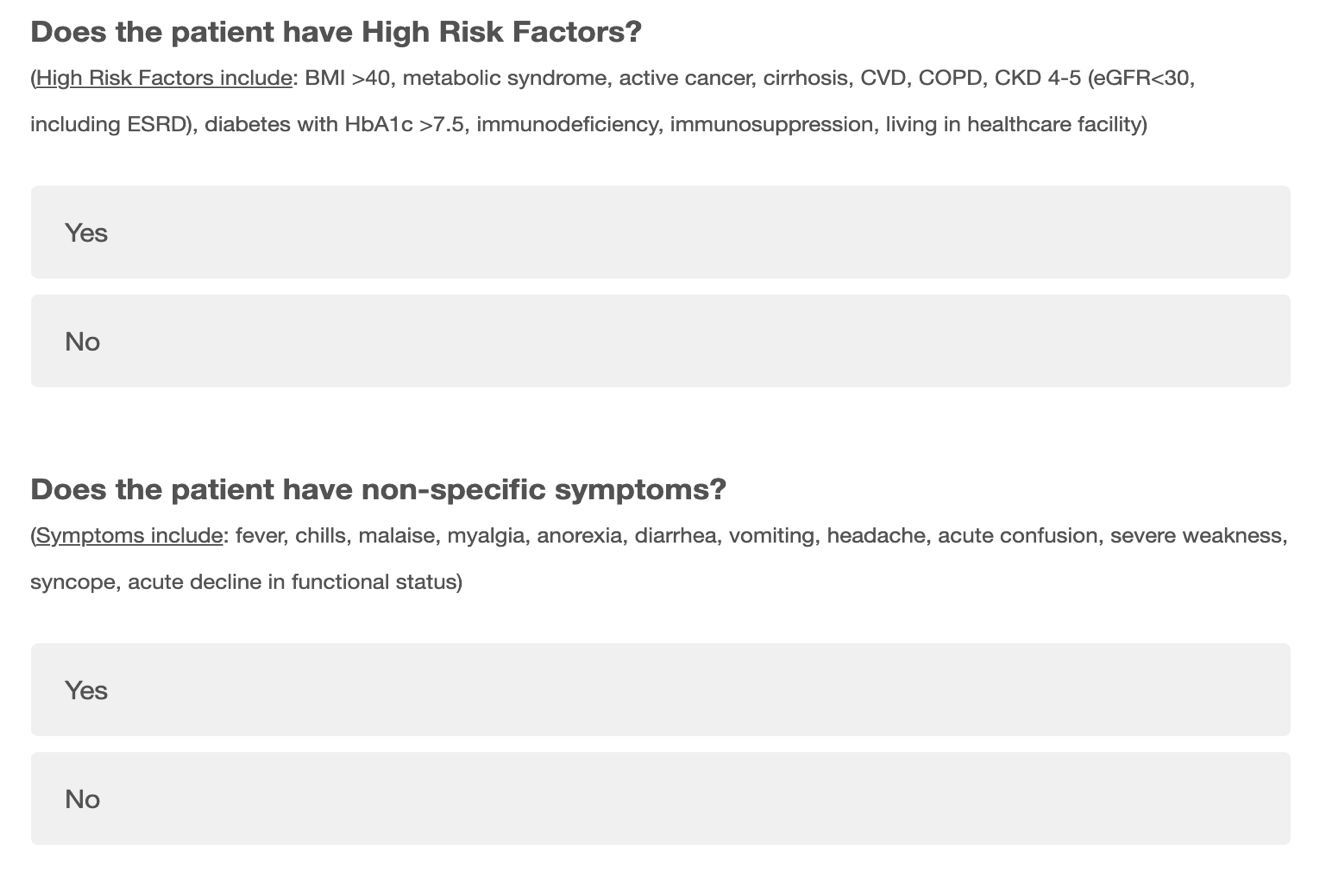


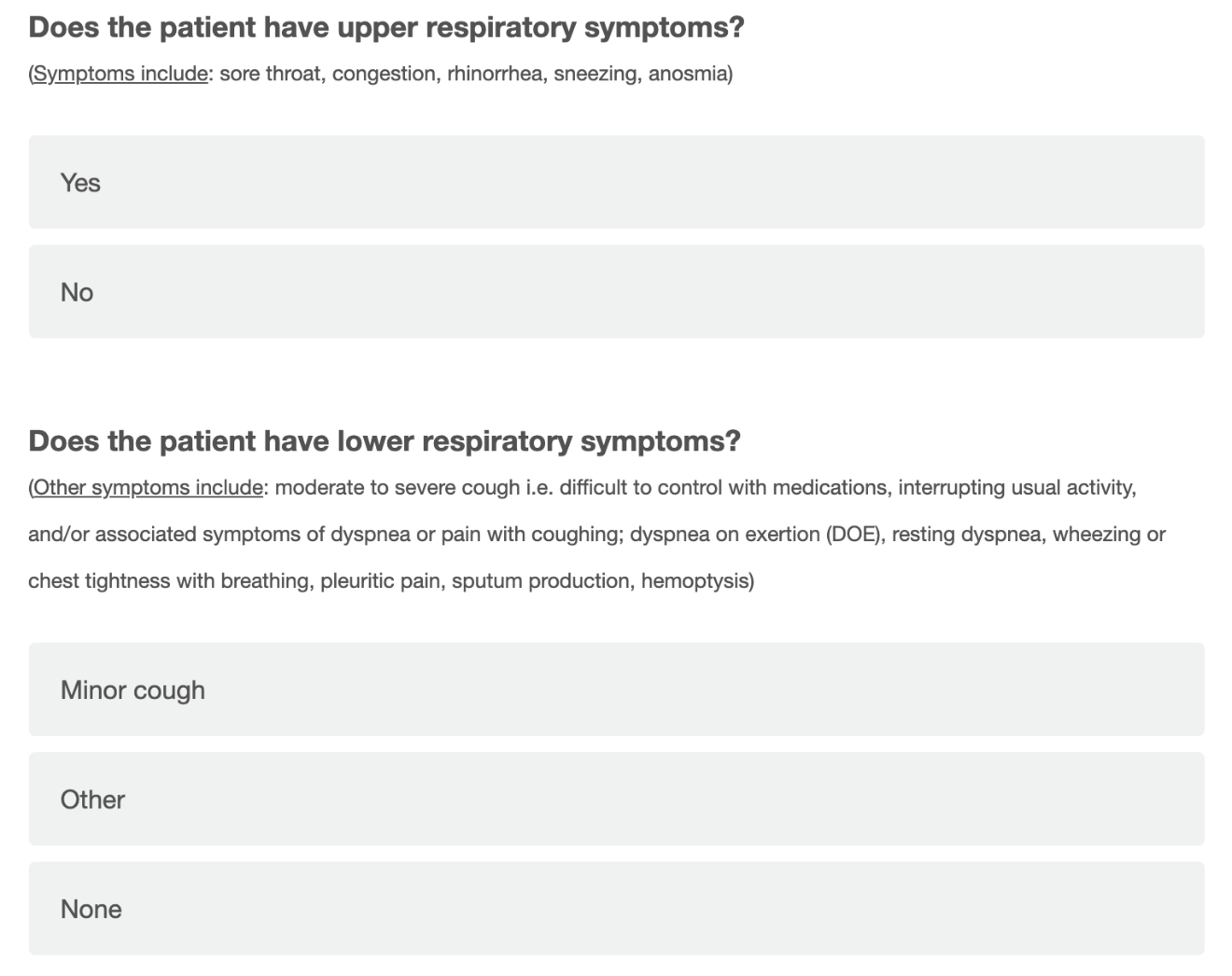


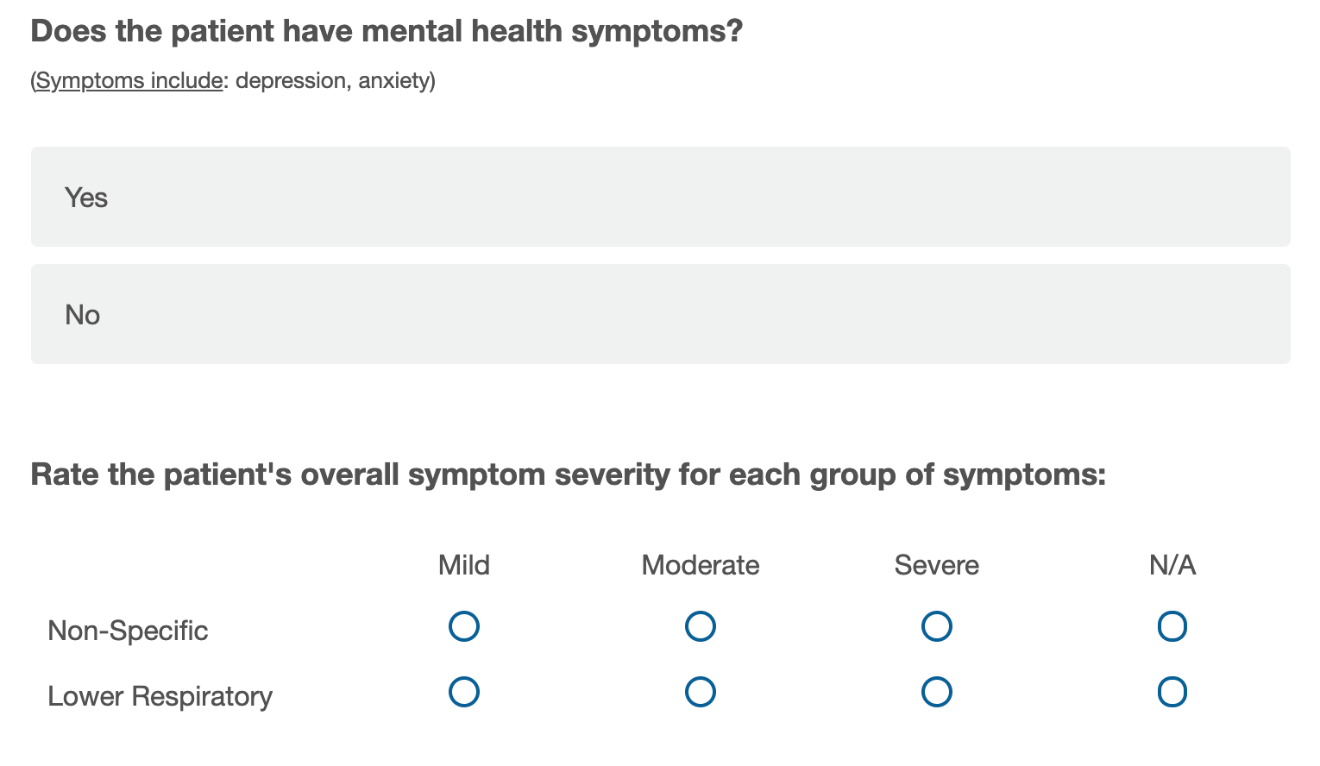


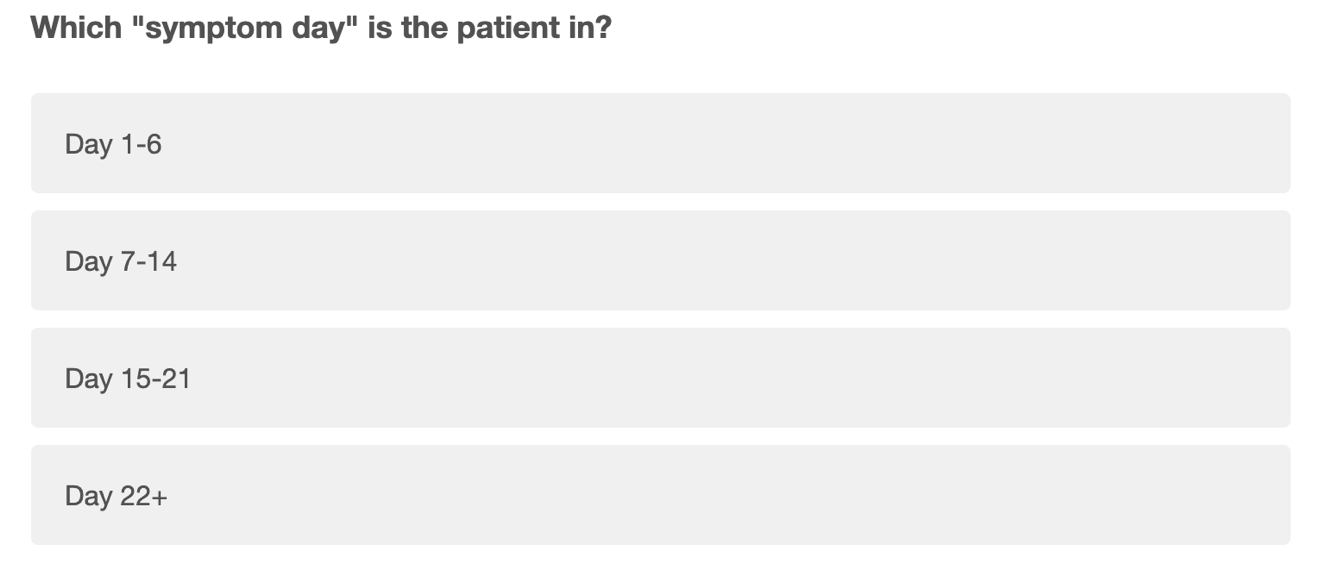


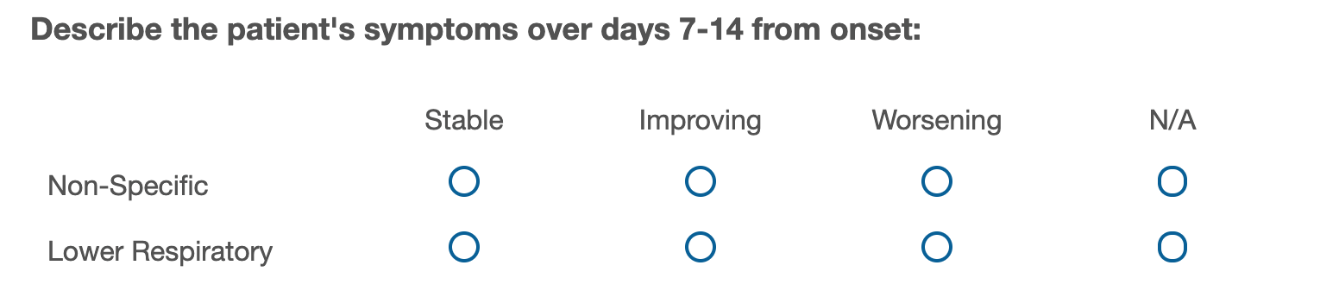


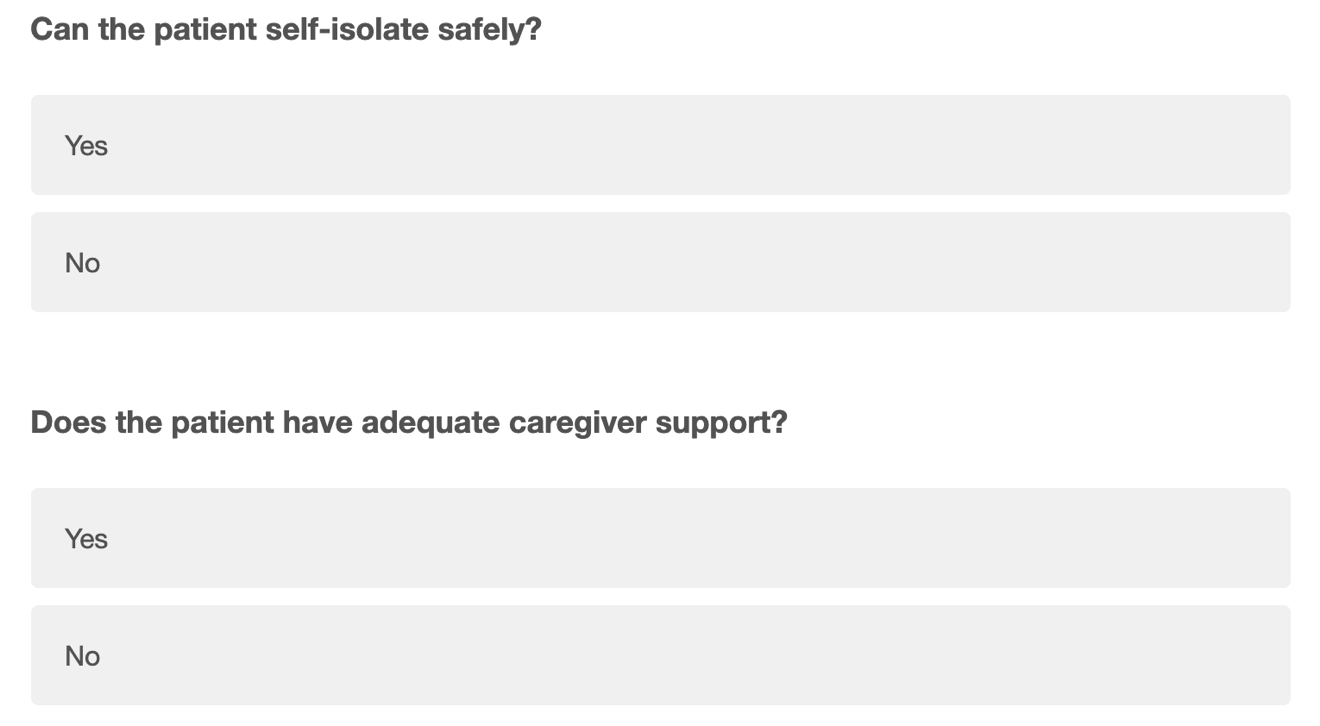

Supplement: Multimedia Appendix 1 [file formative_v7i1e44250_app1.docx]
